# Rhizospheric-Derived *Nocardiopsis alba* BH35 as an Effective Biocontrol Agent Actinobacterium with Antifungal and Plant Growth-Promoting Effects: In Vitro Studies

**DOI:** 10.4014/jmb.2301.01001

**Published:** 2023-02-16

**Authors:** Mohamed H. El-Sayed, Abd El-Nasser A. Kobisi, Islam A. Elsehemy, Mohamed A. El-Sakhawy

**Affiliations:** 1 Department of Botany and Microbiology, Faculty of Science, Al-Azhar University, Cairo 11884, Egypt; 2 Plant Protection Department, Desert Research Center, Cairo, Egypt; 3 Department of Natural and Microbial Products Chemistry, Division of Pharmaceutical and Drug Industries Research, National Research Center, Cairo, Egypt; 4 Department of Medical Laboratory Sciences, College of Applied Medical Sciences, Prince Sattam bin Abdulaziz University, Al-Kharj 11942, Saudi Arabia; 5 Department of Medicinal and Aromatic Plants, Desert Research Center, Cairo, Egypt

**Keywords:** Rhizosphere, *Nocardiopsis alba* BH35, antifungal, plant growth-promoting, biocontrol

## Abstract

The biocontrol approach using beneficial microorganisms to control crop diseases is becoming an essential alternative to chemical fungicides. Therefore, new and efficient biocontrol agents (BCA) are needed. In this study, a rhizospheric actinomycete isolate showed unique and promising antagonistic activity against three of the most common phytopathogenic fungi, *Fusarium oxysporum* MH105, *Rhizoctonia solani* To18, and *Alternaria brassicicola* CBS107. Identification of the antagonistic strain, which was performed according to spore morphology and cell wall chemotype, suggested that it belongs to the *Nocardiopsaceae*. Furthermore, cultural, physiological, and biochemical characteristics, together with phylogenetic analysis of the 16S rRNA gene (OP869859.1), indicated the identity of this strain to *Nocardiopsis alba*. The cell-free filtrate (CFF) of the strain was evaluated for its antifungal potency, and the resultant inhibition zone diameters ranged from 17.0 ± 0.92 to 19.5 ± 0.28 mm for the tested fungal species. Additionally, the CFF was evaluated in vitro to control *Fusarium* wilt disease in *Vicia faba* using the spraying method under greenhouse conditions, and the results showed marked differences in virulence between the control and treatment plants, indicating the biocontrol efficacy of this actinomycete. A promising plant-growth promoting (PGP) ability in seed germination and seedling growth of *V. faba* was also recorded in vitro for the CFF, which displayed PGP traits of phosphate solubilization (48 mg/100 ml) as well as production of indole acetic acid (34 μg/ml) and ammonia (20 μg/ml). This study provided scientific validation that the new rhizobacterium *Nocardiopsis alba* strain BH35 could be further utilized in bioformulation and possesses biocontrol and plant growth-promoting capabilities.

## Introduction

Matching supply with demand as the world population rapidly increases is one of the most significant challenges for the agriculture field. The loss of crop yield due to phytopathogens (bacterial, fungal, pests, nematodes, etc.), especially various fungal phytopathogens, is reaching catastrophic levels. Globally, around 20%of economically important crops are lost due to plant diseases caused by pathogens, and 85% of these losses are caused by fungi [1]. Recent literature suggests that using contemporary chemical pesticides is harmful to human health, the environment, and plant growth [2]. Some of these adverse reactions include the appearance of resistant pathogens, soil acidification, groundwater contamination, destruction of ecosystems, and toxicity to humans and livestock [3]. The devastating effects of pesticides on the habitat has led to their use being restricted in several countries [4]. This ban further necessitates the quest for biological control as an effective and long-lasting alternative to control various soilborne fungal diseases [5]. The specificity to the host plant, adaptiveness of most biocontrol agents to the environment, involvement of several mechanisms of disease suppression by a single microorganism, and complex organismal interactions contribute to the belief that biological control is more effective and durable than chemical fungicides [6].

Microbial antagonists are widely used for the biocontrol of fungal plant diseases instead of chemical fungicides [7]. Numerous microorganisms live in the portion of soil modified or influenced by a plant’s roots and known as the rhizosphere. Microbes that make up the active part of this rhizosphere also affect the soil and growth of the plant in numerous ways [8]. Actinomycetes are aerobic, spore-forming gram-positive bacteria with high GC content (guanine-cytosine content) known to constitute a large part of the rhizosphere microbiota in various environments. Among prokaryotes, actinomycetes are the most beneficial bacteria biologically and economically, as they produce biologically active substances such as antibiotics and enzymes that improve soil health [9].

Evidence indicates that actinomycetes are quantitatively and qualitatively crucial in the rhizosphere, where they may influence plant growth and protect plant roots against invasion by root pathogenic fungi [10]. The antagonistic activity of actinobacteria against phytopathogens is related to the production of antimicrobial compounds [11] and extracellular hydrolytic enzymes capable of lysing fungal cell walls [12]. Additionally, they promote plant growth by producing indole-3-acetic acid to help root growth, siderophores to improve nutrient uptake [13], and several other secondary metabolites as antibiotics [14]. Antagonism from actinomycetes against many phytopathogenic fungi has been reported in many genera [15-17], including the genus *Nocardiopsis* [18-20].

Members of the genus *Nocardiopsis* belong to the phylum Actinomycetota, class Actinobacteria, order Actinomycetales and family *Nocardiopsaceae* [21]. They are gram-positive, aerobic, nonacid-fast, nocardioform bacteria having both substrate and aerial mycelia with long chains of spores. Species of the genus *Nocardiopsis*, like most other actinomycetes, are generally found in soil. Different types of soil samples (saline, hypersaline, desert, and alkaline) have yielded several novel isolates [22]. A large number of species have been reported from various habitats, such as salterns, desert soils, marine environments, and the rhizosphere of plants [23-25].

The genus *Nocardiopsis* is an important genus of actinomycetes due to its substantial application in many fields, such as agriculture [26], environmental protection [27], and industry [28], especially considering its potential to develop novel natural products [22]. Moreover, it is one of the main genera of actinomycetes whose antifungal activity in vitro has been investigated to control phytopathogenic fungi [29], such as soil *Nocardiopsis dassonvillei* against *Bipolaris sorokiniana* in wheat [18], airborne *Nocardiopsis alba* to control *Ganoderma boninense* [19], and soil *Nocardiopsis* sp. to control *Fusarium* sp. [30].

The use of actinobacteria as a biocontrol and plant growth-promoting agent for improving soil and plant health has become an attractive strategy for developing sustainable agricultural systems due to their eco-friendliness, low production cost, and minimal consumption of nonrenewable resources [16, 31]. Therefore, finding actinomycetes strains with such biological activities is of great significance, and to our knowledge, such activities have not been reported previously in the rhizosphere soil-isolated *N. alba*. The present study investigated *N. alba* strain BH35, isolated from rhizospheric soil in the Baqaa Governorate in Hail Province, Kingdom of Saudi Arabia, for its dual activity as an antagonistic and plant growth-promoting actinobacterium. Furthermore, this study is of great significance as it will aid in exploring the antifungal potential of the extracellular metabolites produced by the soil-inhabiting *Nocardiopsis* sp., which can then be exploited for future use as an eco-friendly and cost-effective bio fungicide or BCA with PGP traits.

## Materials and Methods

### Soil Sampling and Treatment

A total of twenty-seven rhizospheric sandy soils (approx. 100 g each) were sampled from three different localities representing three governorates in Hail Province, Kingdom of Saudi Arabia. The geographical locations and sample characteristics of the collected soils are recorded in Table S1. The soil samples were taken from the root area at a depth of 10-15 cm after removing 3 cm of the soil surface. The samples were placed into sterile polythene bags, sealed tightly, and then kept in an icebox at approximately 4°C. The collected soils were sieved to exclude the large particles followed by air-drying at 45°C for 5 h, heat treatment at 70°C for 20 min, and then mixing with 1%CaCO_3_ for 24 h [32].

### Isolation of Actinomycetes

Soil suspensions were prepared from the pretreated soils by mixing 1.0 g soil with 100 ml of sterile distilled water, followed by incubation under shaking conditions at 30°C and 200 rpm for 30 min [33]. For isolation of actinomycetes, serial dilutions up to 10^-7^ were performed from the prepared suspensions, and then 0.1 ml of the final dilution was spread over the surface of inorganic salt starch agar (International *Streptomyces* Project No. 4, ISP–4) medium containing (g/l) soluble starch 10.0, K_2_HPO_4_ 1.0, MgSO_4_·7H_2_O 1.0, NaCl 1.0, (NH_4_)_2_SO_4_ 2.0, CaCO_3_·2H_2_O 2.0, at pH 7.2 ± 0.2. All plates were incubated at 30°C for 7 days. The grown actinomycete colonies were then picked up, purified, maintained on the same media slants at 4°C, and finally stored in 20% glycerol under refrigerated conditions.

### Fungal Test Strains

The fungal species used in this study included three culture collection strains (*Candida albicans* ATCC 10231, *Aspergillus niger* ATCC 16404, and *Aspergillus flavus* ATCC 16883) obtained from the Agriculture and Biology Research Division, National Research Centre, Cairo, Egypt, as well as three of the most common phytopathogenic strains (*Fusarium oxysporum* MH105, *Rhizoctonia solani* To18 and *Alternaria brassicicola* CBS107) from the Plant Protection Department, Desert Research Center, Cairo, Egypt. All fungal test strains were cultured on potato dextrose agar (PDA: 39 g/l Merck, Germany) plates, maintained on the same media slants, and then stored at 4°C for further tests.

### Screening for Antifungal Activity from the Isolated Actinomycetes

The purified actinomycetes isolates were cultured on ISP–4 plates, incubated at 30–33°C for 7 days, and then tested for antagonistic activity against the standard and phytopathogen fungal test strains using agar plug/culture disc diffusion according to the method of Crawford *et al*. [34] with a slight modification. Briefly, agar plugs (6 mm in diameter) taken from the plated actinomycetes were placed on the surface of the cultured PDA fungal test strains, and the plates were then incubated at 25 ± 2°C for 4 days. The formed inhibition zone (IZ) around each plug was measured (mm) and recorded.

To confirm its antifungal activity, the most potent isolate was further tested against the phytopathogenic fungal strains by dual culture plate assay, according to the modified method of Dikin *et al*. [35]. Briefly, the actinomycete culture was streaked in a single line in the middle of 90 mm PDA plates, and a 3 mm mycelium disk from the fungus was then placed on each side of the same plate with a distance of 2.0 cm between the actinomycete line and fungus agar plug. The plates were then incubated for 7 days at 25 ± 2°C. The antifungal assays were performed in triplicate.

### Identification of the Most Active Actinomycete Isolate BH35

**Cultural and Morphological Characteristics**

The cultural characteristics of isolate BH35 were studied by following the guidelines adopted by Shirling and Gottlieb [36]. Visual observation of the culture characteristics, including colors of aerial mass, substrate mycelium, and diffusible pigments, was recorded after cultivation on seven ISP media at 30°C for 7 days.

The morphological characteristics of isolate BH35 grown on ISP–2 (yeast extract–malt extract agar) medium at 30°C for 7 days were studied according to Bergey’s Manual of Systematic Bacteriology [37, 38]. The spore-bearing hyphae and their entire spore chains were investigated under a light microscope (400×) using the coverslip culture technique. For visualization of the spore surface, the cells were fixed in 2.5% glutaraldehyde for 1 h, refixed in 2%osmium tetroxide, and then dehydrated with ethanol. The dehydrated sample was coated with gold-palladium and investigated at different magnifications with scanning electron microscopy (SEM) [39].

### Chemotaxonomic Analyses

Colonies in 7-day-old cultures of the isolate BH35 were scraped from three ISP–2 plates and processed for the isomers of diaminopimelic acid (LL-DAP or *meso*-DAP) and whole-cell sugar pattern analyses. Diaminopimelic acid detection was carried out by paper chromatography of the total cell hydrolysate in 6 N HCl based on the method described by Becker *et al*. [40]. The whole cell sugar pattern was determined by thin-layer chromatography of cells hydrolyzed in 1 N H_2_SO_4_ according to the method of Lechevalier and Lechevalier [41].

### Physiological and Biochemical Characteristics

Following the methods of Goodfellow [42] and Holt *et al*. [43], the physiological and biochemical characteristics of actinomycete isolate BH35 were established. The characteristics determined were utilization of different carbon and nitrogen sources, ability to grow at different temperatures (10–70°C), pH values (5–12), NaCl concentrations (1-6%), and various concentrations of chemical inhibitors; crystal violet (0.0001%), potassium tellurite (0.001%), sodium azide (0.01%), and phenol (0.1%). Moreover, the isolate has the ability to hydrolyze protein, lipid, starch, and elastin, in addition to nitrate reduction and H_2_S production capacity. Entire testing of the physiological and biochemical characteristics was conducted at 33 ± 2°C and 7 days of incubation.

### 16S rRNA Nucleotide Sequencing and Phylogenetic Analyses

**DNA Isolation and PCR-Amplification of the 16S rRNA Gene**

A five-day-old culture of isolate BH35 grown on ISP–2 media was used to inoculate 50 ml of ISP–2 broth and incubated at 200 rpm and 33°C for 72 h to form a pellet of vegetative cells (presporulation). The extraction of total genomic DNA was conducted following the methods described by Sambrook *et al*. [44].

The 16S rRNA gene was amplified by PCR using a GeneAMP PCR System 9700 from PE Applied Biosystems (Perkin Elmer, USA) using the universal primers F27, 5’-AGAGTTTGATCMTGGCTCAG-3’ and R1492 5’-TACGGYTACCTTGTTACGACTT-3’ according to the method described by Edwards *et al*. [45]. Amplification was conducted for 30 cycles of 1 min at 94°C, 1 min of annealing at 53°C, and 2 min of extension at 72°C. The PCR mixture was then analyzed via agarose gel electrophoresis, and the remaining mixture was purified using QIAquick PCR purification reagents (Qiagen, USA).

### Nucleotide Sequencing and Phylogenetic Analyses

The 16S rRNA gene was sequenced on both strands via the dideoxy chain termination method [46]. The 16S rDNA gene (1.5 kb) sequence of the PCR product was acquired using a Terminator Cycle Sequencing Kit (ABI Prism 310 Genetic Analyzer, Applied Biosystems, USA). The obtained sequences were matched with previously published bacterial 16S rRNA sequences in the National Center for Biotechnology Information (NCBI) database using the GenBank BLAST search tool available through the center’s website (http://www.ncbi.nlm.nih.gov/BLAST). Sequence alignment was performed by the CLUSTAL W program. The phylogenetic tree was inferred using the neighbor-joining method with bootstrap testing (1,000 replicates), and the evolutionary distances were computed using the Jukes-Cantor method in MEGA11 [47].

### Production of Active Metabolites and Preparation of CFF

Fresh seed (five-day-old) culture of the isolate BH35 was scraped from an ISP–2 plate, inoculated into 75 ml of ISP–2 broth medium in a 250 ml conical flask and incubated in a rotary shaker at 150 rpm for 72 h at 33°C. Then, a 500 ml flask containing 150 ml of production (starch nitrate broth, SNB) medium (g/l: starch 20, KNO_3_ 2, K_2_HPO_4_ 1, MgSO_4_·7H_2_O 0.5, NaCl 0.5, CaCO_3_ 2, FeSO_4_·7H_2_O 0.01, and distilled water up to 1 L) at pH 7.0 ± 0.2 was inoculated with (10%, v/v) of the cultured seeds and incubated for 7 days at 33°C with shaking at 150 rpm. After fermentation, the culture broth was prefiltered using a cotton layer, filtered with a Whatman filter (0.45 μm), and finally centrifuged at 10,000 ×*g* for 30 min at 4°C. The prepared CFF was stored at −20°C for the following investigations.

### Assay of Antifungal Activity of CFF

To assay the antifungal activity of the CFF of isolate BH35, agar-well diffusion was performed according to the method of Niño *et al*. [48] with a slight modification. Briefly, the fungal strains were subcultured on PDA plates at 25 ± 2°C for 4 days. A loopful of each culture was suspended individually in 10.0 ml of sterile saline (aqueous 0.45% NaCl), and the turbidity was adjusted to give a suspension of approximately 10^7^-10^8^ spores/ml. A 1.0 ml sample of each fungal saline suspension was used to inoculate a 500 ml conical flask containing 250 ml of PDA. Wells (6 mm) were cut in the inoculated plates using a sterile cork borer, and 100 μl of the CFF was transferred to each well. The plates were left for 2 h at 4°C and then incubated at 25 ± 2°C for 4 days. After incubation, the resultant IZ was measured (mm) and recorded.

### Investigation of the Damage Effect Caused by CFF on the Fungal Mycelium

To examine the effect of the CFF on the fungal structure and mycelium formation, the most sensitive fungal test strain, *F. oxysporum* MH105, was cultured on PDA plates inoculated individually with the effective concentrations (75 and 100%) of the CFF and incubated at 25 ± 2°C for 4 days. Fungal agar plugs (6 mm in diameter) were cut from each plate and fixed by placing them in vials containing 2% paraformaldehyde in 0.1 M sodium cacodylate buffer (pH 7.2) and 3% glutaraldehyde at 4°C for 4 h. These samples were then washed three times with 0.1 M sodium cacodylate buffer (pH 7.2) for 10 min. Subsequently, the fixed samples were dehydrated using a graded ethanol series 70-100% for 10 min and observed with SEM.

### Evaluation of CFF as a Biological Control for *Fusarium* Wilt in Planta

To evaluate the disease control effect of the actinomycete BH35 in the agricultural field, the CFF of the strain was tested against *F. oxysporum* MH105 wilt in *V. faba* planta using the spraying method under greenhouse conditions [49]. The fungal suspension was prepared by inoculating 3 discs of *F. oxysporum* MH105 grown in PDA into 150 ml potato dextrose broth medium and incubated at 25 ± 2°C and 150 rpm for 10 days. To collect the formed mycelia, the culture content was mixed with an electric mixer and centrifuged at 600 ×*g* for 10 min. Seedlings of *V. faba* (7 days old) planta were sprayed with the prepared suspension (10^5^ conidia/ml) until runoff occurred. After inoculation, the plants were covered with closed transparent plastic bags for 48 h in a greenhouse under controlled conditions (with a 14 h photoperiod at 25°C).

Using a Preval sprayer, leaves and stems of the infected *V. faba* planta at the beginning of day 3 of the infection were sprayed individually with 70 and 100% CFF (10 ml/each treatment group). All sides and surfaces of the planta were sprayed until runoff was ensured. The control planta was sprayed with uninoculated SNB as described above. All plants were incubated at 25°C for 10 days, examined daily to record the changes that occurred, and photographs were taken after 3, 7, and 10 days of inoculation. The experiments were performed in triplicate.

### Assay of PGP Activity of CFF

**Effect of CFF on Seed Germination**

Seeds of three economic plants, bread wheat (*Triticum sativum*), barley (*Hordeum vulgare*), and beans (*Vicia faba*), obtained from the Agriculture Research Center, Cairo, Egypt, were used in this study. Bioassays used Petri dishes (90 mm diameter) with one sheet of Whatman no. 1 filter paper as the substrate. The filtrate of isolate BH35 was diluted to different concentrations of 25, 50, 75, and 100% using distilled water. Seeds of the selected plants were surface sterilized using sodium hypochlorite (0.3% v/v) for 10–12 min and washed three times in sterile double-distilled water. The seeds were placed on Whatman no. 1 filter paper. An aliquot (5 ml) from each concentration was used for treatments; sterile noninoculated SNB was used as a control. After adding seeds and aqueous solutions, Petri dishes were sealed with parafilm and incubated in the dark at 25°C. After growth, plants were frozen at -10°C for 24 h to avoid subsequent development during the measurement process [50].

**Effect of CFF on Seedling Growth**

To confirm the PGP capability of isolate BH35, the bean plant, as the most promoted plant, was selected for testing the promotion of seedling growth. In each treatment, a total of ten bean seeds were fully submerged for 12 h in 10 ml of the CFF. The treated seeds were placed into a plug tray (9 × 7 × 6 cm) filled with sterilized soil (180 g each), with three seedlings in each tray. Seeds submerged in sterile noninoculated SNB were used as the negative control. The biometric properties (germination percentage, shoot length, and root length) were measured on day 10 after seed sowing, and this experiment was repeated three times [51].

### Bioassay of Compounds Involved in PGP Activity

**Indole Acetic Acid (IAA) Production**

The actinomycetes isolate BH35 was grown on ISP-2 medium at 30°C for five days. Six-millimeter diameter agar discs were cut using a sterile cork borer and inoculated into 100 ml of ISP-2 broth containing 0.2% L-tryptophan. The culture was incubated at 30°C with continuous shaking at 125 rpm for 7 days in the dark. After incubation, the suspension was centrifuged at 11,000 ×*g* for 15 min. The produced IAA, expressed in μg/ml, was assessed using the Salkowski colorimetric assay [52].

### Phosphate Solubilization

The phosphate solubilization test was conducted qualitatively by inoculating the potential actinomycete isolate BH35 on phosphate agar medium containing Ca_3_(PO_4_)_2_. A halo clearing zone around the growing colony after incubation at 28°C for seven days was used as an indicator for positive P solubilization [53]. The ability of this isolate to solubilize insoluble phosphate was also tested quantitatively in liquid culture. The tested isolate was inoculated into a phosphate medium containing 10 g of tricalcium phosphate, 10 g of MgCl_2_·6H_2_O, 5 g of MgSO_4_·7H_2_O, 0.25 g of KCl, and 0.2 g of (NH_4_)_2_SO_4_. The culture was incubated at 30°C for seven days. The culture suspensions were centrifuged at 3,000 ×*g* for 30 min. Soluble phosphate in the supernatants was determined as previously described by Hamdali *et al*. [54].

### Ammonia Production

The actinomycetes isolate BH35 was tested for ammonia production using the method described by Cappuccino and Sherman [55]. In this method, 20 μl of seed culture was propagated in 10 ml of peptone water and incubated at 30°C with shaking at 120 rpm for 15 days. Subsequently, 0.5 ml of Nessler's reagent was added to the culture, and the development of a brown to yellow color indicated a positive test for ammonia production. The absorbance was measured at 530 nm using a Thermo Scientific spectrophotometer, compared with the standard curve of (NH_4_)_2_SO_4_, and expressed in mg/ml.

### Statistical Analysis

Data collected from the above experiments are the mean values of three independent replicates. The data were subjected to analysis of variance (ANOVA) using the statistical package SPSS v17. The comparison between treatments was analyzed using the Tukey HSD test at a significance level of *p* ≤ 0.05.

## Results

### Antifungal Activity from Rhizospheric-Isolated Actinomycetes

**Fig. 1:**
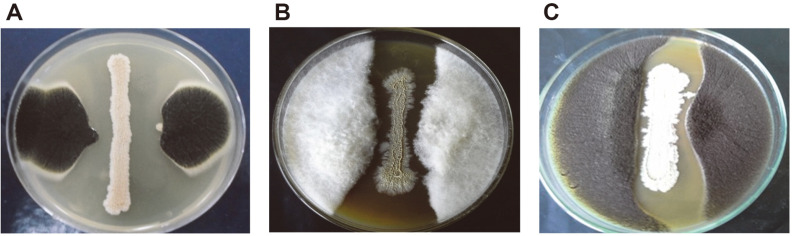
Antagonistic activity of actinomycete isolate BH35 against phytopathogenic fungal species using dual culture assay on PDA plates. (**A**) *Rhizoctonia solani* To18. (**B**) *Fusarium oxysporum* MH105. (**C**) *Alternaria brassicicola* CBS107.

A total of thirty-nine rhizospheric actinomycete cultures of different morphological appearances were isolated from three governorates in Hail Province, Kingdom of Saudi Arabia, and screened for their antifungal activities against standard and phytopathogenic fungal species (Table S2). Only twelve isolates (30.76%) exhibited varying degrees of antagonistic activity against the tested strains. Among them, isolate BH35, which was isolated from the Baqaa Governorate, was the most promising, exhibiting a broad antifungal spectrum against the tested fungi. For the standard fungal strains, the inhibition activity was recorded against *C. albicans* ATCC 10231, *A. niger* ATCC 16404, and *A. flavus* ATCC 168833 with mean IZ diameters of 13.6 ± 0.28, 15.8 ± 0.41, and 17.1 ± 0.36 mm, respectively. For the phytopathogenic fungal species, the mean IZ diameters were 22.3 ± 0.51, 20.4 ± 0.40, and 18.8± 0.76 mm against *F. oxysporum* MH105, *R. solani* To18, and *A. brassicicola* CBS107, respectively. As a result, the actinomycete BH35 was selected as the most potent isolate and further retested by the streaking method against the same phytopathogenic fungal species (Fig. 1).

### Identification of Actinomycete Isolate BH35

**Conventional Identification**

**Fig. 2:**
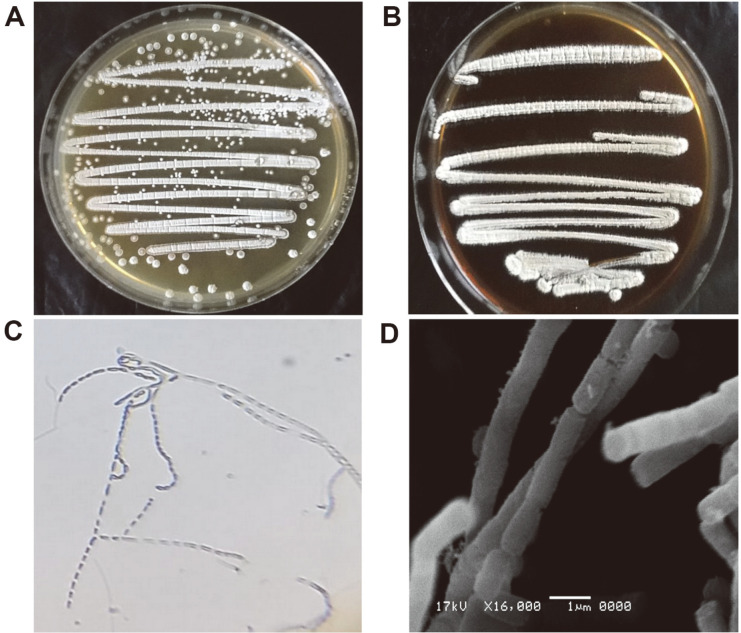
Cultural and morphological characteristics of actinomycete isolate BH35. (**A**) White aerial mycelium formed on ISP–4 medium. (**B**) Strong brown-melanin pigment produced on ISP–6 medium. (**C**) Aerial hyphae bearing zigzag-shaped spore chains under light microscopy (400×). (**D**) SEM micrograph (16,000×) showing recti-flexible chains of rod-shaped spores with a smooth surface.

The cultural characteristics of isolate BH35 grown on 7–ISP media were recorded (Table S3) and showed moderate to abundant growth for the organism on all tested media except ISP–3, which revealed only weak growth, and ISP–7, which showed no growth. The color of the mature aerial mycelium was white (Fig. 2A) on all ISP media except ISP–1, which was light gray. The substrate mycelium varied from light yellow to dark yellowish brown. Melanin pigment was produced only on ISP–6 medium (Fig. 2B), and no other diffusible pigments were recorded for the organism on any of the tested media. The morphological characteristics of BH35 grown on ISP–2 medium were studied by light microscopy (400×), which demonstrated zigzag-shaped aerial hyphae (Fig. 2C), and SEM (16,000×), which revealed recti-flexible chains of rod-shaped spores with a smooth surface (Fig. 2D).

Chemotaxonomic analyses of whole-cell hydrolysates of the isolate BH35 showed that the strain has *meso*-2,6-diaminopimelic acid (*meso*-DAP), and no diagnostic sugars were detected, indicating that the cell wall peptidoglycan belongs to type III (Wall-chemo type III); thus, BH35 has the exact composition of symbolic cellular components as the genus *Nocardiopsis*. The results of physiological and biochemical tests concluded that abundant growth was recorded for BH35 when grown on fructose, D-glucose, and sucrose, as well as L-asparagine and L-tryptophan as sole carbon and nitrogen sources, respectively. The organism succeeds in growing at a wide range of pH values (5–11, with optimum growth at 10), temperature (20–45°C, optimum at 33°C), and NaCl (1–4%, optimum at 3%). The obtained results of other physiological and biochemical tests are recorded in Table 1.

### Molecular Identification

**Fig. 3:**
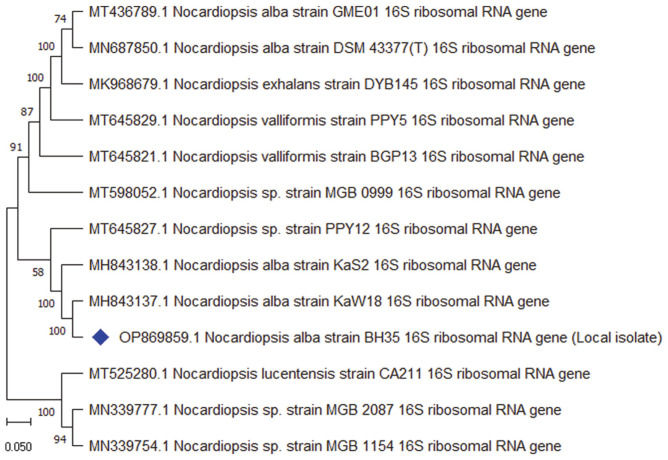
Phylogenetic tree of *Nocardiopsis alba* strain BH35 inferred using the neighbor-joining method in MEGA 11.0 software. This tree shows the relationships between isolate BH35 and closely linked species of the genus *Nocardiopsis*. The bootstrap consensus tree inferred from 1,000 replicates is taken to represent the evolutionary history of the taxa analyzed. The percentage of replicate trees in which the associated taxa clustered together in the bootstrap test (1,000 replicates) are shown next to the branches. This analysis involved 13 nucleotide sequences. All ambiguous positions were removed for each sequence pair (pairwise deletion option). There were a total of 1,752 positions in the final dataset.

Classical identification of the actinomycete isolate BH35 was confirmed by nucleotide sequencing of the 16S rRNA gene (1,470 bp), which was submitted to the GenBank database under accession no. OP869859.1. The phylogenetic tree inferred using the neighbor-joining method (Fig. 3) shows the relationships between BH35 and closely linked species of the genus *Nocardiopsis*. The evolutionary distances were computed using the Jukes-Cantor method and are in units of the number of base substitutions per site in 13 nucleotide sequences. The results of the phylogenetic analyses revealed that BH35 shared 100% 16S rRNA gene sequence similarity with *N. alba* strain KaW18 (GenBank accession no. MH843137.1); therefore, it was assigned the name *N. alba* strain BH35.

### Antifungal Effects of CFF of *N. alba* Strain BH35

The promising actinomycete *N. alba* BH35 was cultivated on SNB medium scale (3 L), and the culture fermentation broth was collected and filtered to obtain the CFF (2.3 L total volume), which was then kept at -20°C for further investigations. The CFF of *N. alba* BH35 was tested for its antifungal activity against the phytopathogenic fungal species using the agar-well diffusion method. The recorded antagonistic activities were similar to those obtained from the agar plug/culture disc method, where the resultant IZ diameters were 19.5 ± 0.28, 18.6 ± 0.32, and 17.0 ± 0.92 mm for *F. oxysporum* MH105, *R. solani* To18, and *A. brassicicola* CBS107, respectively.

**Fig. 4:**
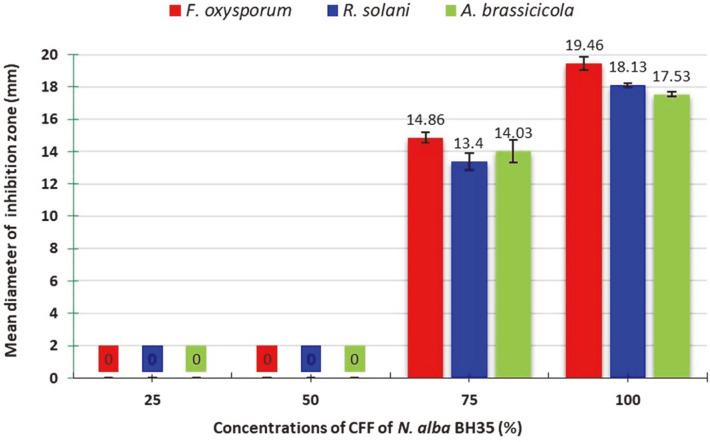
Inhibition ability of the different concentrations of *N. alba* BH35 CFF against phytopathogenic fungal species. The values represent the mean ± SD of triplicate experiments (*n* = 3, *p* < 0.05).

To confirm the antagonistic potential of *N. alba* BH35, different concentrations ranging from 25 to 100% of the CFF of BH35 were tested against the same phytopathogenic fungal species. The results presented in Fig. 4 show that the CFF suppressed the radial growth of all phytopathogenic strains at concentrations of 75 and 100% with IZ diameters ranging from 13.4 ± 0.53 – 14.86 ± 0.31 mm to 17.53 ± 0.15 – 19.46 ± 0.42 mm, respectively. However, it was inactive at lower concentrations.

**Fig. 5:**
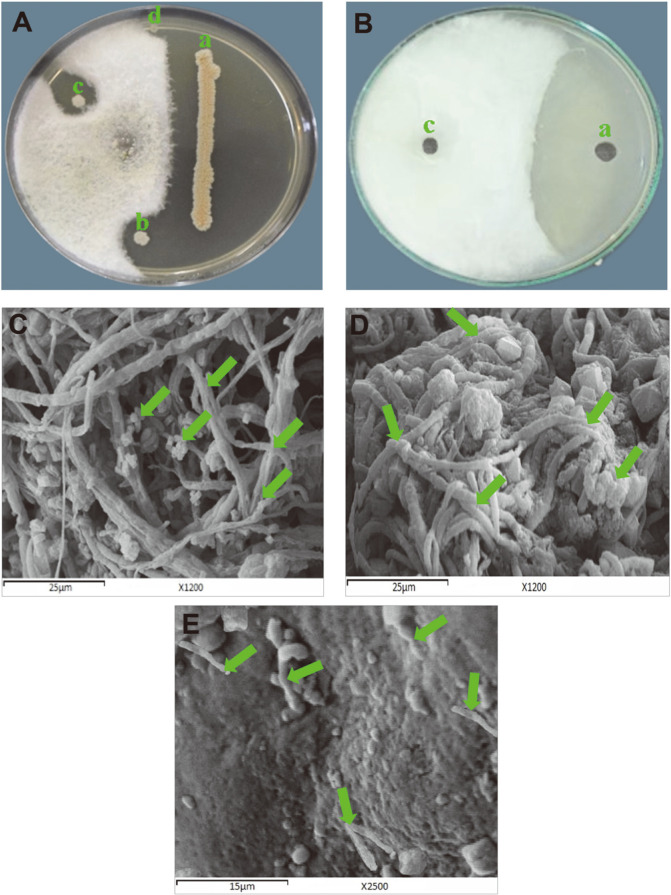
Antifungal effects of CFF of *N. alba* BH35 against phytopathogenic *F. oxysporum*. (**A**) Antagonistic effect using a dual culture assay on PDA plate. In this plate, the actinomycete was inoculated with gradual inoculum sizes in two different forms, where (a) is a linear very heavy inoculum, and (b, c, d) are circular moderate, mild, and very mild inoculums, respectively. (**B**) Inhibitory effect of CFF (100.0% conc.) against radial growth on Czapek-Dox plate. In this plate, (c) is the control (uninoculated SNB medium), and (a) is the CFF of actinomycete. (**C**) SEM micrograph (1200×) of untreated (control) mycelium. (**D**) SEM micrograph (1200×) of treated mycelium at 70% CFF. (**E**) SEM micrograph (2500×) of treated mycelium at a CFF concentration of 100%.

The abovementioned results of antifungal assays demonstrated that *F. oxysporum* was the most sensitive fungal test strain. To verify the efficacy of *N. alba* BH35 as a potential biocontrol agent against phytopathogenic fungi, especially wilt-mold *F. oxysporum* MH105, both the culture and CFF of *N. alba* BH35 were reassayed with different techniques against *F. oxysporum* MH105 cultured on different growth media (Figs. 5A and 5B). The results confirmed the antagonistic potential that was obtained from the primary screening. To explain the antifungal effect of the CFF on the mycelium structure of *F. oxysporum* MH105, it was treated with effective concentrations of 75.0 and 100% CFF, and then the treated plates were investigated under SEM. The results showed that untreated (control) plates showed normally germinated spores and homogeneous, condensed, intact and viable mycelia (Fig. 5C) when compared to the structural damage shown by shrunken, broken, and compacted mycelia acted upon by a concentration of 75% (Fig. 5D). Meanwhile, the concentration of 100%(Fig. 5E) showed no mycelium formation at all, and only very small fragments of the fungal mycelium.

### Capability of *N. alba* BH35 CFF as Biocontrol for *Fusarium* Wilt in *V. faba*

**Fig. 6:**
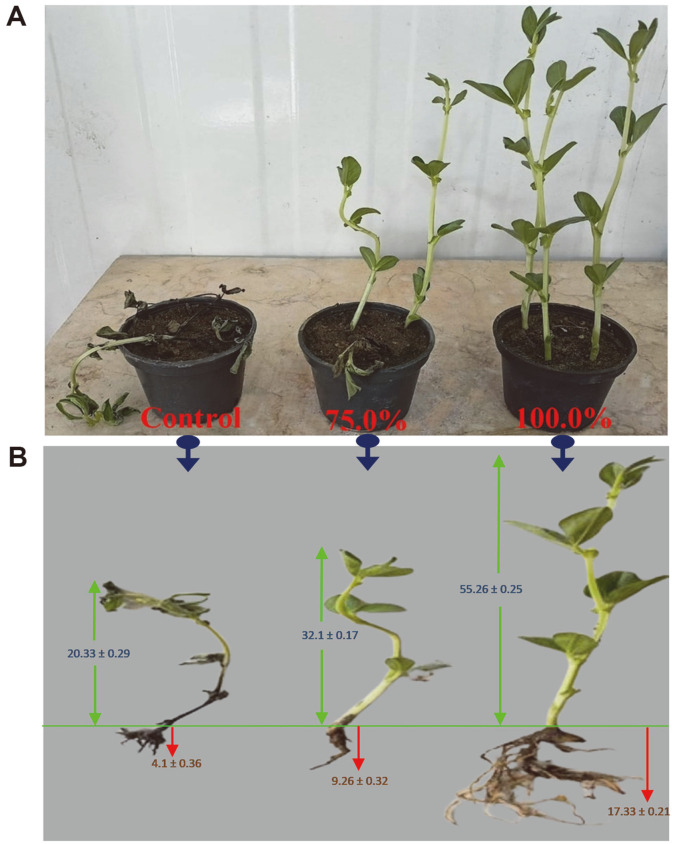
In vitro biocontrol capability of CFF of *N. alba* BH35 against phytopathogenic wilt-mold *F. oxysporum* in *V. faba* under greenhouse conditions. (**A**) Pathogenicity and treatment. (**B**) Suppression effect of the pathogen on the shoot and root length of the infected plants. The values represent the mean ± SD of triplicate experiments (*n* = 3, *p* < 0.05).

The capability of the CFF of *N. alba* BH35 as a biocontrol agent was evaluated in vitro to control wilt-mold *F. oxysporum* MH105 in *V. faba* using the spraying method under greenhouse conditions (Fig. 6). *F. oxysporum* MH105 successfully infected *V. faba* leaves and stems after inoculation of mycelia at 25°C for 10 days. The results of pathogenicity experiments revealed clear differences in virulence between *V. faba* control (receiving SNB medium) and treatment (receiving 70 and 100% CFF) plants (Fig. 6A). In control plants, after 5 days of infection, leaves and stems showed wilting and mild-brownish spots that increased with time; by day 10, all plants were dead. In the treated plants, the outbreak of the pathogen was partially prevented together with the appearance of mild wilting and some brownish spots on some of the infected plants after application of the CFF at a concentration of 70%, while at a concentration of 100%, no symptoms of infection were present, and plants appeared completely healthy. Additionally, one of the most important observations in the pathogenicity experiments was the suppression effect of the pathogen on the growth of the infected plants, where the shoot and root lengths were markedly affected in the control and 70% treatments compared with the 100% CFF treatment (Fig. 6B).

### PGP Effects of *N. alba* Strain BH35 CFF

The CFF of the actinomycete *N. alba* strain BH35 was assessed for its stimulatory effect on seed germination of *T. sativum*, *H. vulgare*, and *V. faba* plants using a filter paper bioassay in Petri dishes (Fig. S1A). The results of bioassays demonstrate that the CFF possesses PGP activity on both shoot and root length of the germinated seeds in a concentration-dependent manner, where it was increased by increasing the CFF concentration (Fig. S1B). We observed obvious differentiations in germination between control (receiving SNB medium) and treatment (receiving concentrations 25-100% CFF) seeds, especially in *V. faba* plants.

### Capability of *N. alba* BH35 CFF as PGP for *V. faba* Seedlings

**Fig. 7:**
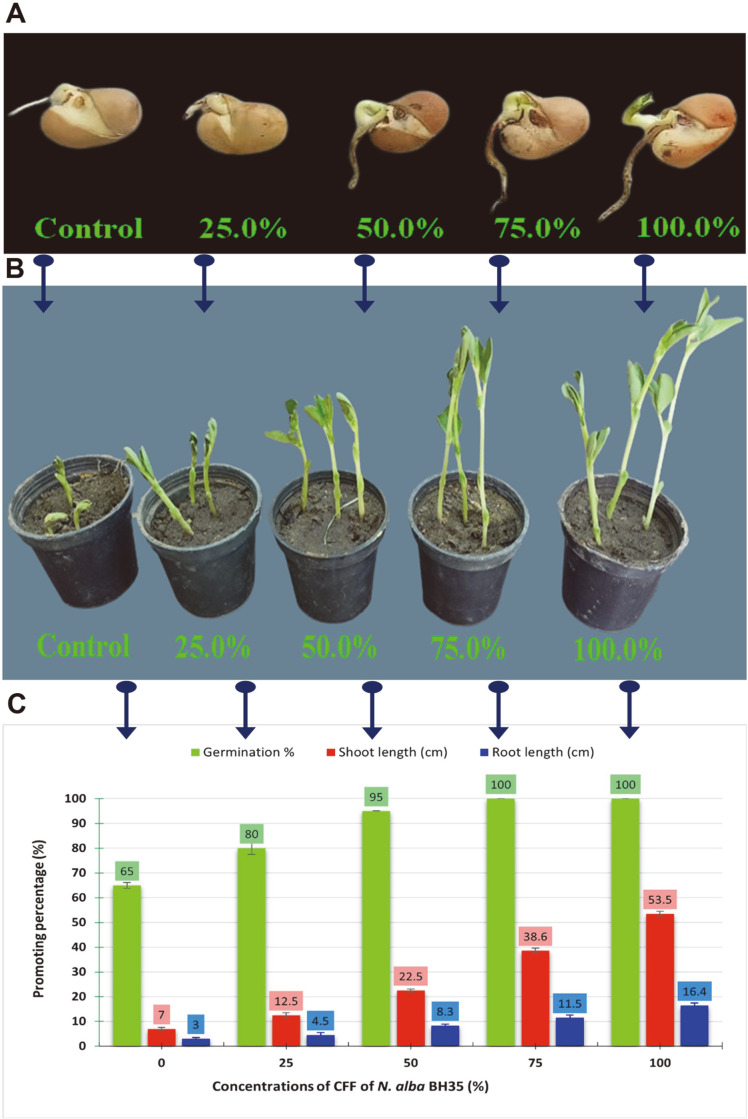
Promoting capability of the different concentrations of *N. alba* BH35 CFF for growth of *V. faba*. (**A**) Seed germination. (**B**) Seedling growth. (**C**) Promotion percentages in seed, shoot, and root length. In this experiment, the control was uninoculated SNB medium. Different concentrations of CFF (25-100%) were prepared by dilution with double distilled water. The values represent the mean ± SD of triplicate experiments (*n* = 3, *p* < 0.05).

The results obtained from the PGP bioassays demonstrated that *V. faba* was the most promoted plant. To confirm the efficacy of *N. alba* BH35 for use as a potential PGP agent, especially for *V. faba*, different concentrations ranging from 25% to 100% CFF were reassessed for their efficacy in promoting the growth of *V. faba* under greenhouse conditions (Fig. 7). The results obtained from this experiment confirmed the findings of the abovementioned bioassays, where the increase in seed germination (Fig. 7A) and seedling growth (Fig. 7B) was concentration dependent. Concentrations of 25% and 50% recorded the lowest promoting effect, while the highest effect was achieved by the concentrations of 75% and 100% CFF (Fig. 7C).

### Characterization of PGP Traits of CFF

*N. alba* BH35 was able to solubilize inorganic phosphate and was identified as a potential phosphate-solubilizing agent based on a clear halo zone around the inoculated culture (Fig. S2). The CFF of BH35 was assessed for its PGP traits, and the results of quantitative estimations demonstrated that the amount of phosphate solubilized was 48 mg/100 ml; IAA production was 34 μg/ml, and the production of ammonia was equal to 20 μg/ml.

## Discussion

Synthetic chemical fungicides have been successfully used in the agricultural sector on a regular basis to control a variety of microbial phytopathogens, especially plant pathogenic fungi. Recently, these agrochemicals have been phased out in many countries due to increased environmental contamination and associated problems with food security [56, 57]. All modern agricultural systems seek to ensure sustainable crop production, so there is a constant requirement for new natural resources and eco-friendly crop protection techniques such as biological control [58].

Currently, microorganism-based biological control is one of the best options for fighting plant pathogens and increasing crop yields without harming the environment. During our screening for effective BCAs from actinomycetes against some phytopathogenic fungi, 12 isolates were among 39 morphologically different actinomycetes isolated from sandy rhizospheric soils, and these isolates exhibited varying antagonistic activity against three of the most common phytopathogenic fungal species, *F. oxysporum* MH105, *R. solani* To18, and *A. brassicicola* CBS107. The use of microorganisms in agriculture is an effective method for controlling fungal phytopathogens, providing long-term protection, and promoting plant growth and health [59, 60]. Microorganisms in soil play vital roles in soil fertility, where they break down organic matter, cycle nutrients, and interact beneficially with plants. One of the major constituents among the soil microbial population is actinomycetes, which are capable of producing many bioactive secondary metabolites, such as antibiotics [61], particularly when they exist in the rhizosphere of plants [62]. These metabolites aid in the defense of plants against a variety of phytopathogens, specifically fungal and bacterial organisms [63]. Our findings from the screening program were similar to those of Chen *et al*. [51], who reported the isolation of 32 actinomycetes with different morphologies from rhizosphere soils. Of them, 16 isolates exhibited various inhibitory degrees against *F. oxysporum*.

Among the antagonistic actinomycete isolates, BH35 was selected as the most potent isolate, with IZ diameters of 22.3 ± 0.51, 20.4 ± 0.40, and 18.8 ± 0.76 mm for *F. oxysporum* MH105, *R. solani* To18, and *A. brassicicola* CBS107, respectively. Finding actinomycete isolates with multiple activities against such destructive phytopathogens is a promising outcome. Therefore, these findings suggest that isolate BH35 could be a multifunctional BCA. To confirm its antagonistic activity, it was further tested by the streaking method against the same phytopathogenic fungal species using a dual culture assay, and it was able to inhibit the radial growth of the tested species to a high and promising degree. The dual culture assay is the most common method used for in vitro screening of microorganisms possessing high-throughput antagonism against fungal pathogens [64].

Many genera of actinomycetes have been documented as BCAs, making them a valuable resource for the biocontrol of fungi-induced plant diseases [15, 17, 19, 20]. In this study, identification of the selected isolate BH35 was performed with polyphasic approaches using different classical and molecular characteristics. The white aerial mycelium shown from the culture characteristics, the zigzag-shaped aerial hyphae demonstrated by the morphological features, and wall-chemo type III recorded from the chemotaxonomic analyses are consistent with some members of the genus *Nocardiopsis*, according to Lechevalier and Lechevalier [41] and Bergey's Manual of Systematic Bacteriology [37, 38]. The physiological and biochemical characteristics coupled with phylogenetic analyses of the 16S rRNA gene sequencing proved that BH35 was identical to the species *N. alba* [38, 43]. Similarly, Abdelrahman *et al*. [61] screened rhizosphere soil-isolated actinomycetes for their metabolic activity against the growth of the fungal plant pathogen *Phytophthora infestans*, some of which were identified as *Nocardiopsis* spp. relying on partial 16S rRNA sequencing.

To confirm the antagonistic potential of *N. alba* BH35, its CFF at different concentrations was further evaluated in vitro for inhibition activity against the same fungal pathogens. These procedures were consistent with those reported in a previous study, wherein 107 soil actinomycetes were tested for antagonistic potential against some of the fungal phytopathogens using the dual-culture assay, and in which the isolate with the most potential was selected, characterized, and further evaluated in vitro for antagonistic activity [65]. Our results from this assay showed that the CFF suppressed the radial growth of the tested fungal pathogens at concentrations of 75 and 100%. Additionally, the results demonstrated that *F. oxysporum* was the most sensitive strain. These results are in agreement with Patel and Thakker [66], who reported marked antagonistic activity of the soil-isolated actinomycete *Nocardiopsis dassonvillei* strain YM12 against *Fusarium* sp., while they were in contrast with Kunova *et al*. [58], who reported inhibition activity of some actinomycete strains against the mycelium growth of 6 soilborne fungal phytopathogens, of which *F. oxysporum* was the least affected compared with the other tested fungi. Different Ascomycota fungi, especially *Fusarium* spp., are soilborne fungal phytopathogens that harm a number of economically significant crops. Within this genus, *Fusarium oxysporum* is one of the most widely distributed pathogens worldwide [67]. This pathogen is known to cause a well-known vascular disease named "*Fusarium* wilt," which results in significant losses of many crops. As a result, agricultural practices such as biological control have been developed to combat this disease.

Although a number of BCAs from different microorganisms, such as *Pseudomonas* spp. [68], *Bacillus* spp. [69], *Trichoderma* spp. [70, 71], *Penicillium* spp. [72], and *Streptomyces* spp. [73, 74], as well as from some species of the genus *Nocardiopsis*, such as *Nocardiopsis dassonvillei* MB22 [18], *Nocardiopsis dassonvillei* strain YM12 [66], *Nocardiopsis* sp. [68], *Nocardiopsis albirubida* strain NA2 [20], *Nocardiopsis* sp. [30], and airborne *N. alba* [19], have been investigated to control *Fusarium* wilt and/or other fungal phytopathogens, none has been reported previously in rhizosphere soil-derived *N. alba*, especially in Saudi Arabia.

SEM was used to investigate the damage effect caused by the CFF of *N. alba* BH35 on mycelium formation in *F. oxysporum* MH105. The results recorded from SEM micrographs showed structural damage in the mycelium explained by shrunken, broken, and compacted hyphae acted upon by the CFF, which exerted its effects in a concentration-dependent manner. Similar results were recorded by Kim *et al*. [75], who reported abnormal, crumpled, and shrunken spores and hyphae of *F. oxysporum* MH105 following treatment with the antifungal extracts of *Streptomyces blastmyceticus* strain 12-6. Based on these data, we predict that the antifungal effect of the CFF studied here is similar to the effect of some antifungal agents, such as *Streptomyces natalensis*-produced natamycin, which binds to ergosterol in fungal cell membranes. We predict that the CFF of strain BH35 has one or more of the active compounds able to inhibit the growth of mycelial fungi; therefore, further investigations are required to identify these antifungal compounds.

We investigated the efficiency of the CFF of *N. alba* BH35 to control *Fusarium* wilt in one of the essential economic crops in Egypt, *V. faba*, using the spraying method under greenhouse conditions. The control plants (sprayed with SNB), leaves and stems showed wilting and mild- brownish spots on the 5^th^ day of infection. These symptoms increased over time, and by the 10^th^ day, all plants were dead. The increase in infection may be attributed to the components of the SNB medium used, which enhanced fungal growth. In the treated plants (sprayed with 70 and 100% CFF), the outbreak of the pathogen was significantly blocked at a concentration of 70%, and brownish spots were also decreased. At a concentration of 100%, no symptoms of infection were present. These results confirm that the CFF of *N. alba* strain BH35 can be sprayed on *V. faba* and other crops to prevent fungal wilt disease, and this actinomycete strain could be further utilized in bioformulation experiments and marketed as a biopesticide or BCA. Our findings are in agreement with those reported by other researchers who have studied biocontrol of *Fusarium* wilt in *V. faba* using *Bacillus* spp. [76], tomato using *S. roseoflavus* NKZ-259 [75], and flax using *Streptomyces* sp. IA1 [77]. The present study is the first report on *N. alba* BH35 as a biocontrol agent of *Fusarium* wilt in *V. faba*.

The second promising outcome of our study is the stimulatory effect recorded by the CFF of *N. alba* BH35 on the seed germination and seedling growth of three economic plants, *T. sativum*, *H. vulgare*, and *V. faba*. This stimulation was a concentration-dependent effect, where it increased with increasing filtrate concentration. Kunova *et al*. [58] reported that it is crucial that any potential BCA does not adversely impact the host plant's germination and development. We did not record any negative effects while applying the filtrate to control *Fusarium* wilt in our experiment; in contrast, our findings demonstrated a positive correlation between the BC and PGP efficiencies of the CFF. Berg [78] reported that BC and PGP are two distinct but related actions associated with healthy plant‒microbe interactions, and they may use similar molecular mechanisms. Moreover, application of this filtrate promoted plant growth of *V. faba* under greenhouse conditions. This promotion was monitored by the increase in root and shoot length of the treatment when compared to the control. Furthermore, displaying different PGP traits of phosphate solubilization and production of indole acetic acid and ammonia increased the viability of our actinomycete strain. Selection of strains with PGP capability requires in vitro assessment of compounds involved in enhancing root and shoot length. Diverse actinomycetes genera are known to produce PGP-related metabolites [13].

The rhizosphere-derived actinomycete *N. alba* strain BH35, which was isolated from the Baqaa Governorate in Hail Province, Kingdom of Saudi Arabia, exhibited remarkable antifungal activity against various destructive phytopathogenic fungal species. Identification of this strain was performed with polyphasic approaches using classical and molecular characteristics. In vitro, under greenhouse conditions, the CFF of this strain succeeded in controlling *Fusarium* wilt disease and promoted plant growth of *V. faba* with promising effects, as well as displayed some PGP traits. Therefore, this strain can be exploited for future use as an eco-friendly and cost-effective biofungicide or BCA with PGP properties. To our knowledge, these activities have not been reported previously in *N. alba*. Due to these capabilities, further investigations are required to isolate and purify the active compounds sharing these effects, and to test their applications in the field.

## Supplemental Materials

Supplementary data for this paper are available on-line only at http://jmb.or.kr.

## Figures and Tables

**Table 1:** Physiological and biochemical characteristics of the isolate, BH35.

Characteristics	Growth result	Characteristics	Growth result
(1) Melanin pigment production		(5) Growth at different temperature (°C)	
Liquid tryptone yeast extract	+	20	+
Tyrosine agar	−	30	++
Peptone yeast extract iron agar	+	33	+++
(2) Utilization of carbon sources		40 – 45	Wg
Fructose	+++	50	−
D-glucose	+++	(6) Tolerance to NaCl (%)	
D-mannitol	++	1 – 3	+++
L-arabinose	−	4	Wg
Inositol	−	5	−
L-rhamnose	−	(7) Degradation ability	
D-xylose	Wg	Citrate	++
Sucrose	+++	Pectin	+
Cellulose	+	Tyrosine	−
Dextrin	Wg	Esculin	Wg
Glycerol	++	Xanthine	−
(3) Utilization of nitrogen sources		Urea	+
L-tyrosine	−	(8) Tolerance to growth inhibitors (%)	
L-lysine	−	Crystal violet (0.0001)	+
L-arginine	++	Potassium tellurite (0.001)	−
L-asparagine	+++	Sodium azide (0.01)	+
L-cysteine	−	Phenol (0.1)	−
L-histidine	+	(9) Sensitivity to antibiotics (μg)	
L-phenylalanine	+	Gentamicin (30)	S
L-tryptophan	+++	Rifampicin (50)	S
(4) Growth at different pH values		Vancomycin (50)	R
5	Wg	Streptomycin (50)	R
6	+	Penicillin G (10 IU)	R
7	+	(10) Other biochemical tests	
8	++	Nitrate reduction	+
9	++	H_2_S production	−
10	+++	Amylase	+
11	+	Catalase	+
12	−	Lipase	+

“−” Negative, “Wg” weak growth, “+” Moderate, “++” Good, “+++” Abundant growth, “R” Resistant, “S” Sensitive.

## References

[1] Oerke EC (2006). Crop losses to pests. J. Agric. Sci..

[2] Rani L, Thapa K, Kanojia N, Sharma N, Singh S, Grewal AS (2021). An extensive review on the consequences of chemical pesticides on human health and environment. J. Clean. Prod..

[3] Bhardwaj T, Sharma JP (2013). Impact of pesticides application in agricultural industry: an Indian scenario. Int. J. Agric. Food Sci. Technol..

[4] Hamblin JD (2015). Pick your poison Banned: a history of pesticides and the science of toxicology Frederick Rowe Davis Yale University Press, 2014. pp. 284.. Science.

[5] Laxmishree C, Nandita S (2017). Botanical pesticides, a major alternative to chemical pesticides: a review. Int. J. Life Sci..

[6] Kaur T, Rani R, Manhas RK (2019). Biocontrol and plant growth promoting potential of phylogenetically new *Streptomyces* sp. MR14 of rhizospheric origin. AMB Express.

[7] De Vrije T, Antoine N, Buitelaar RM, Bruckner S, Dissevelt M, Durand A (2001). The fungal biocontrol agent *Coniothyrium minitans*: production by solid-state fermentation, application and marketing. Appl. Microbiol. Biotechnol..

[8] Sokolova TA (2015). Specificity of soil properties in the rhizosphere: analysis of literature data. Eurasian Soil Sci..

[9] Bhatti AA, Haq S, Bhat RA (2017). Actinomycetes benefaction role in soil and plant health. Microb. Pathog..

[10] Poomthongdee N, Duangmal K, Pathom-Aree W (2015). Acidophilic actinomycetes from rhizosphere soil: diversity and properties beneficial to plants. J. Antibiot. (Tokyo).

[11] Daquioag JEL, Penuliar GM (2021). Isolation of actinomycetes with cellulolytic and antimicrobial activities from soils collected from an urban green space in the Philippines. Int. J. Microbiol..

[12] Mukherjee G, Sen SK (2006). Purification, characterization, and antifungal activity of chitinase from *Streptomyces venezuelae* P10. Curr. Microbiol..

[13] Khamna S, Yokota A, Lumyong S (2009). Actinomycetes isolated from medicinal plant rhizosphere soils: diversity and screening of antifungal compounds, indole-3-acetic acid and siderophore production. World J. Microbiol. Biotechnol..

[14] Shi L, Nwet TT, Ge B, Zhao W, Liu B, Cui H (2018). Antifungal and plant growth-promoting activities of *Streptomyces roseoflavus* strain NKZ-259. Biol. Control.

[15] El-Tarabily KA, Soliman MH, Nassar AH, Al-Hassani HA, Sivasithamparam K, McKenna F (2000). Biological control of *Sclerotinia minor* using a chitinolytic bacterium and actinomycetes. Plant Pathol..

[16] Ebrahimi-Zarandi M, Saberi Riseh R, Tarkka MT (2022). Actinobacteria as effective biocontrol agents against plant pathogens, an overview on their role in eliciting plant defense. Microorganisms.

[17] Le KD, Yu NH, Park AR, Park DJ, Kim CJ, Kim JC (2022). *Streptomyces* sp. AN090126 as a biocontrol agent against bacterial and fungal plant diseases. Microorganisms.

[18] Allali K, Goudjal Y, Zamoum M, Bouznada K, Sabaou N, Zitouni A (2019). *Nocardiopsis dassonvillei* strain MB22 from the Algerian Sahara promotes wheat seedlings growth and potentially controls the common root rot pathogen *Bipolaris sorokiniana*. J. Plant Pathol..

[19] Widada J, Damayanti E, Alhakim MR, Yuwono T, Mustofa M (2021). Two strains of airborne *Nocardiopsis alba* producing different volatile organic compounds (VOCs) as biofungicide for *Ganoderma boninense*. FEMS Microbiol. Lett..

[20] Allali K, Zamoum M, Benadjila A, Zitouni A, Goudjal Y (2022). Optimisation of talcum powder formulation based on *Nocardiopsis albirubida* strain NA2 spores for biocontrol of *Bipolaris sorokiniana* root rot in durum wheat seedlings. Biocontrol Sci. Technol..

[21] Sun H, Lapidus A, Nolan M, Lucas S, del Rio TG, Tice H (2010). Complete genome sequence of *Nocardiopsis dassonvillei* type strain (IMRU 509 T). Stand. Genomic Sci..

[22] Bennur T, Kumar AR, Zinjarde S, Javdekar V (2015). *Nocardiopsis* species: incidence, ecological roles and adaptations. Microbiol. Res..

[23] Chun J, Kyung Sook Bae, Eun Young Moon, Jung SO, Hong Kum Lee, Kim SJ (2000). *Nocardiopsis kunsanensis* sp. nov., a moderately halophilic actinomycete isolated from a saltern. Int. J. Syst. Evol. Microbiol..

[24] Fang C, Zhang J, Pang H, Li Y, Xin Y, Zhang Y (2011). *Nocardiopsis flavescens* sp. nov., an actinomycete isolated from marine sediment. Int. J. Syst. Evol. Microbiol..

[25] Zhang YG, Lu XH, Ding YB, Zhou XK, Wang HF, Guo JW (2016). *Nocardiopsis rhizosphaerae* sp. nov., isolated from rhizosphere soil of Halocnermum strobilaceum (Pall.) Bieb. Int. J. Syst. Evol. Microbiol..

[26] AbdElgawad H, Zinta G, Abuelsoud W, Hassan YM, Alkhalifah DHM, Hozzein WN (2021). An actinomycete strain of *Nocardiopsis lucentensis* reduces arsenic toxicity in barley and maize. J. Hazard. Mater..

[27] Patel GB, Rakholiya P, Shindhal T, Varjani S, Tabhani NM, Shah KR (2021). Lipolytic *Nocardiopsis* for reduction of pollution load in textile industry effluent and SWISS model for structural study of lipase. Bioresour. Technol..

[28] Adenan NH, Lim YY, Ting ASY (2021). *Nocardiopsis* sp. for the removal of triphenylmethane dyes: decolorization and optimization studies. Water. Air. Soil Pollut..

[29] Torres-Rodriguez JA, Reyes-Pérez JJ, Quiñones-Aguilar EE, Hernandez-Montiel LG (2022). Actinomycete potential as biocontrol agent of phytopathogenic fungi: mechanisms, source, and applications. Plants (Basel).

[30] Adlin Jenifer JSC, Michaelbabu M, Eswaramoorthy Thirumalaikumar CL, Jeraldin Nisha SR, Uma G, Citarasu T (2019). Antimicrobial potential of haloalkaliphilic *Nocardiopsis* sp. AJ1 isolated from solar salterns in India. J. Basic Microbiol..

[31] Sathya A, Vijayabharathi R, Gopalakrishnan S (2017). Plant growth-promoting actinobacteria: a new strategy for enhancing sustainable production and protection of grain legumes. 3 Biotech.

[32] Seong CN, Choi JH, Baik KS (2001). An improved selective isolation of rare actinomycetes from forest soil. J. Microbiol..

[33] Sineva O, Terekhova L (2015). Selective isolation of rare actinomycetes from soil. Antibiot. Chemother..

[34] Crawford DL, Lynch JM, Whipps JM, Ousley MA (1993). Isolation and characterization of actinomycete antagonists of a fungal root pathogen. Appl. Environ. Microbiol..

[35] Dikin A, Sijam K, Kadir J, Semanz IA (2006). Antagonistic bacteria against *Schizophyllum commune* Fr. in Peninsular Malaysia. Biotropia.

[36] Shirling EB, Gottlieb D (1966). Methods for characterization of *Streptomyces* species. Int. J. Syst. Bacteriol..

[37] Williams ST, Goodfellow M, Alderson G, Williams ST, Sharpe ME, Holt JP (1989). Genus *Nocardiopsis* Meyer 1976. Bergey's Manual of Systematic Bacteriology Vol. 4, 1st Ed.

[38] Goodfellow M, Kämpfer P, Busse HJ, Trujillo ME, Suzuki K, Ludwig W (2012). Bergey's Manual of Systematic Bacteriology, Volume 5: The Actinobacteria, part A.

[39] Tresner HD, Davies MC, Backus EJ (1961). Electron microscopy of *Streptomyces* spore morphology and its role in species differentiation. J. Bacteriol..

[40] Becker B, Lechevalier MP, Gordon RE, Lechevalier HA (1964). Rapid differentiation between *Nocardia* and *Streptomyces* by paper chromatography of whole-cell hydrolysates. Appl. Microbiol..

[41] Lechevalier MP, Lechevalier H (1970). Chemical composition as a criterion in the classification of aerobic actinomycetes. Int. J. Syst. Bacteriol..

[42] Goodfellow M (1971). Numerical taxonomy of some nocardioform bacteria. J. Gen. Microbiol..

[43] Holt J, Krieg N, Sneath P, Staley J, Williams S (1994). Bergey's Manual of Determinative Bacteriology.

[44] Sambrook J, Maccallum P, Russell D (1989). Molecular Cloning: A Laboratory Manual, 2nd Ed.

[45] Edwards U, Rogall T, Blöcker H, Emde M, Böttger EC (1989). Isolation and direct complete nucleotide determination of entire genes. Characterization of a gene coding for 16S ribosomal RNA. Nucleic Acids Res..

[46] Sanger F, Nicklen S, Coulson AR (1977). DNA sequencing with chain-terminating inhibitors. Proc. Natl. Acad. Sci. USA.

[47] Tamura K, Stecher G, Kumar S (2021). MEGA11: molecular evolutionary genetics analysis version 11. Mol. Biol. Evol..

[48] Niño J, Espinal CM, Mosquera OM, Correa YM (2003). Antimycotic activity of 20 plants from Colombian flora. Pharm. Biol..

[49] Degani O, Maor R, Hadar R, Sharon A, Horwitz BA (2004). Host physiology and pathogenic variation of *Cochliobolus heterostrophus* strains with mutations in the G protein alpha subunit, CGA1. Appl. Environ. Microbiol..

[50] Macías FA, Castellano D, Molinillo JMG (2000). Search for a standard phytotoxic bioassay for allelochemicals. Selection of standard target species. J. Agric. Food Chem..

[51] Chen J, Hu L, Chen N, Jia R, Ma Q, Wang Y (2021). The biocontrol and plant growth-promoting properties of *Streptomyces alfalfae* XN-04 revealed by functional and genomic analysis. Front. Microbiol..

[52] Bric JM, Bostock RM, Silverstone SE (1991). Rapid in situ assay for indoleacetic acid production by bacteria immobilized on a nitrocellulose membrane. Appl. Environ. Microbiol..

[53] Nautiyal CS (1999). An efficient microbiological growth medium for screening phosphate solubilizing microorganisms. FEMS Microbiol. Lett..

[54] Hamdali H, Bouizgarne B, Hafidi M, Lebrihi A, Virolle MJ, Ouhdouch Y (2008). Screening for rock phosphate solubilizing actinomycetes from Moroccan phosphate mines. Appl. Soil Ecol..

[55] Cappuccino J, Sherman N (1992). Microbiology: A Laboratory Manual.

[56] Geissbühler H, Brenneisen P, Fischer HP (1982). Frontiers in crop production: chemical research objectives. Science.

[57] van Lenteren JC, Bolckmans K, Köhl J, Ravensberg WJ, Urbaneja A (2018). Biological control using invertebrates and microorganisms: plenty of new opportunities. BioControl.

[58] Kunova A, Bonaldi M, Saracchi M, Pizzatti C, Chen X, Cortesi P (2016). Selection of *Streptomyces* against soil borne fungal pathogens by a standardized dual culture assay and evaluation of their effects on seed germination and plant growth. BMC Microbiol..

[59] Fernando WGD, Ramarathnam R, Krishnamoorthy AS, Savchuk SC (2005). Identification and use of potential bacterial organic antifungal volatiles in biocontrol. Soil Biol. Biochem..

[60] Albarracín Orio AG, Brücher E, Ducasse DA (2016). A strain of *Bacillus subtilis* subsp. *subtilis* shows a specific antagonistic activity against the soil-borne pathogen of onion *Setophoma terrestris*. Eur. J. Plant Pathol..

[61] Abdelrahman O, Yagi S, El Siddig M, El Hussein A, Germanier F, De Vrieze M (2022). Evaluating the antagonistic potential of actinomycete strains isolated from Sudan's soils against *Phytophthora infestans*. Front. Microbiol..

[62] Hata EM, Sijam K, Ahmad ZAM, Yusof MT, Azman NA NA (2015). In vitro antimicrobial assay of actinomycetes in rice against *Xanthomonas oryzae* pv. *oryzicola* and as potential plant growth promoter. Brazilian Arch. Biol. Technol..

[63] Hasegawa S, Meguro A, Shimizu M, Nishimura T, Kunoh H (2006). Endophytic actinomycetes and their interactions with host plants. Actinomycetologica.

[64] Pliego C, Ramos C, de Vicente A, Cazorla FM (2011). Screening for candidate bacterial biocontrol agents against soilborne fungal plant pathogens. Plant Soil.

[65] Marimuthu S, Karthic C, Mostafa AA, Mohammed Al-Enazi N, Abdel-Raouf N, Nageh Sholkamy E (2020). Antifungal activity of *Streptomyces* sp. SLR03 against tea fungal plant pathogen *Pestalotiopsis theae*. J. King Saud Univ. Sci..

[66] Patel KB, Thakker JN (2019). Growth promotion and biocontrol activity of *Nocardiopsis dassonvillei* strain YM12: an isolate from coastal agricultural land of Khambhat. Vegetos.

[67] Baayen RP, O'Donnell K, Bonants PJM, Cigelnik E, Kroon LPNM, Roebroeck EJA (2000). Gene genealogies and AFLP analyses in the *Fusarium oxysporum* complex identify monophyletic and nonmonophyletic formae speciales causing wilt and rot disease. Phytopathology.

[68] Djebaili R, Pellegrini M, Bernardi M, Smati M, Kitouni M, Gallo M Del (2020). Biocontrol activity of actinomycetes strains against fungal and bacterial pathogens of *Solanum lycopersicum* L. and *Daucus carota* L.: in vitro and in planta antagonistic activity.

[69] Luo W, Liu L, Qi G, Yang F, Shi X, Zhao X (2019). Embedding *Bacillus velezensis* NH-1 in microcapsules for biocontrol of cucumber *Fusarium* wilt. Appl. Environ. Microbiol..

[70] Nieto-Jacobo MF, Steyaert JM, Salazar-Badillo FB, Vi Nguyen D, Rostás M, Braithwaite M (2017). Environmental growth conditions of *Trichoderma* spp. Affects indole acetic acid derivatives, volatile organic compounds, and plant growth promotion. Front. Plant Sci..

[71] Díaz-Gutiérrez C, Arroyave C, Llugany M, Poschenrieder C, Martos S, Peláez C (2021). *Trichoderma asperellum* as a preventive and curative agent to control *Fusarium* wilt in *Stevia rebaudiana*. Biol. Control.

[72] Li Y, Guo Q, Wei X, Xue Q, Lai H (2019). Biocontrol effects of *Penicillium griseofulvum* against monkshood (*Aconitum carmichaelii* Debx.) root diseases caused by *Sclerotium rolfsiii* and *Fusarium* spp. J. Appl. Microbiol..

[73] Peng C, An D, Ding WX, Zhu YX, Ye L, Li J (2020). Fungichromin production by *Streptomyces* sp. WP-1, an endophyte from *Pinus dabeshanensis*, and its antifungal activity against *Fusarium oxysporum*. Appl. Microbiol. Biotechnol..

[74] Heinsch SC, Hsu SY, Otto-Hanson L, Kinkel L, Smanski MJ (2019). Complete genome sequences of *Streptomyces* spp. isolated from disease-suppressive soils. BMC Genomics.

[75] Kim YJ, Kim J heon, Rho JY (2019). Antifungal activities of *Streptomyces blastmyceticus* strain 12-6 against plant pathogenic fungi. Mycobiology.

[76] Haddoudi I, Cabrefiga J, Mora I, Mhadhbi H, Montesinos E, Mrabet M (2021). Biological control of *Fusarium* wilt caused by *Fusarium equiseti* in *Vicia faba* with broad spectrum antifungal plant-associated *Bacillus* spp. Biol. Control.

[77] Toumatia O, Yekkour A, Goudjal Y, Riba A, Coppel Y, Mathieu F (2015). Antifungal properties of an actinomycin D-producing strain, *Streptomyces* sp. IA1, isolated from a Saharan soil. J. Basic Microbiol..

[78] Berg G (2009). Plant-microbe interactions promoting plant growth and health: perspectives for controlled use of microorganisms in agriculture. Appl. Microbiol. Biotechnol..

