# Supplementary material for: Rhizospheric-Derived Nocardiopsis alba BH35 as an Effective Biocontrol Agent Actinobacterium with Antifungal and Plant Growth-Promoting Effects: In Vitro Studies
Source: J Microbiol Biotechnol. 2023 Feb 16;33(5):607–20. doi: 10.4014/jmb.2301.01001 (PMC10236170; doi:10.4014/jmb.2301.01001)
Supplement: Supplementary file 1 [file jmb-33-5-607-supple.pdf]

Table S1. Geographical locations of sampling sites and properties of the collected soils.

Location	Site No.	GPS		Sample code	Number of samples	Sample characteristics
		Latitude (N)	Longitude (E)			
Hiqrusayn Ash Shiqiq	1	27°43'00’N	41°55'03’E	HAS-S1	2	Yellow, sandy, dry, soft soil
		27°43'09’N	41°55'33’E	HAS-S2	3	
		27°43'14’N	41°55'12’E	HAS-S3	3	
Tourbah Hail	2	28°15'37’N	42°55'20'E	TH-S1	4	Yellow, sandy, dry, rough soil
		28°15'33’N	42°55'15'E	TH-S2	3	
		28°15'14’N	42°54'47'E	TH-S3	2	
Baqaa	3	27°53'33’N	42°24'57'E	B-S1	3	Yellowish black, sandy, high moisture, soft soil
		27°53'45’N	42°24'39'E	B-S2	3	
		27°56'51’N	42°23'22'E	B-S3	4	

Table S2. The isolated actinomycetes and their antifungal activities.

Location-Site No.	Isolate code	Colony morphology			Mean diameter (mm) of inhibition zone of antifungal activity					
					Culture collection strains			Phytopathogens species		
		Colour	Consistency	Pigment	*C. albicans* ATCC 10231	*A. niger* ATCC 16404	*A. flavus* ATCC 16883	*F. oxysporum* MH105	*R. solani* To18	*A. brassicicola* CBS107
**1**—S1	HAS1	Grey	Rough	None	0.0	0.0	0.0	0.0	0.0	0.0
	HAS2	Grey	Rough	Red	0.0	12.0±0.32	14.0±0.25	0.0	0.0	0.0
	HAS3	White	Smooth	Brown	0.0	0.0	0.0	0.0	0.0	0.0
**1**—S2	HAS4	Creamy	Rough	Red	0.0	0.0	0.0	0.0	0.0	0.0
	HAS5	Grey	Smooth	None	14.5±0.50	17.0±0.90	15.1±1.2	0.0	0.0	0.0
	HAS6	Grey	Smooth	None	0.0	0.0	0.0	0.0	0.0	0.0
	HAS7	Grey	Smooth	Orange	0.0	0.0	0.0	0.0	0.0	0.0
	HAS8	Grey	Rough	None	0.0	13.8±1.04	0.0	0.0	0.0	0.0
**1**—S3	HAS9	White	Rough	None	0.0	0.0	0.0	0.0	0.0	0.0
	HAS10	Grey	Rough	None	0.0	0.0	0.0	0.0	0.0	0.0
	HAS11	Grey	Rough	Blue	12.5±0.86	14.3±0.76	0.0	14.1±0.32	0.0	0.0
	HAS12	Purple	Smooth	Red	0:0	0.0	15.5±0.50	18.4±0.45	16.1±0.65	15.0±0.50
	HAS13	Creamy	Rough	None	0.0	0.0	0.0	0.0	0.0	0.0
	HAS14	Creamy	Smooth	None	0.0	0.0	0.0	0.0	0.0	0.0
**2**—S1	TH15	Grey	Smooth	Orange	16.0±0.32	0.0	0:0	15.3±0.76	15.6±1.04	0:0
	TH16	Grey	Smooth	Brown	0.0	0.0	0.0	0.0	0.0	0.0
**2**—S2	TH17	White	Rough	None	0.0	0.0	0.0	0.0	0.0	0.0
	TH18	Grey	Smooth	None	0.0	0.0	0.0	0.0	0.0	0.0
	TH19	Grey	Rough	None	0.0	0.0	0.0	0.0	0.0	0.0
	TH20	White	Smooth	None	0.0	0.0	0.0	0.0	0.0	0.0
**2**—S3	TH21	Grey	Smooth	None	0.0	0.0	0.0	0.0	0.0	0.0
	TH22	Grey	Rough	Violet	0.0	18.1±0.28	19.0±0.50	12.7±0.25	12.8±0.76	14.4±0.60
	TH23	Purple	Rough	None	0.0	0.0	0.0	0.0	0.0	0.0
	TH24	Grey	Rough	None	0.0	0.0	0.0	0.0	0.0	0.0
	TH25	Grey	Smooth	None	0.0	0.0	0.0	0.0	0.0	0.0
**3**—S1	BH26	Flesh	Smooth	None	0.0	0.0	0.0	0.0	0.0	0.0
	BH27	Purple	Rough	None	0.0	0.0	0.0	0.0	0.0	0.0
**3**—S2	BH28	Grey	Rough	None	15.2±0.92	0.0	0.0	13.6±1.25	14.3±0.41	0.0
	BH29	White	Rough	None	0.0	0.0	0.0	0.0	0.0	0.0

	BH30	Yellow	Rough	Brown	20.8±0.76	13.3±1.5	12.5±0.60	12.5±0.58	12.9±0.45	12.1±0.28
	BH31	Yellow	Rough	None	0.0	0.0	0.0	0.0	0.0	0.0
	BH32	White	Rough	Orange	23.8±1.04	18.9±0.55	21.5±0.50	17.8±1.04	0.0	0.0
	BH33	Yellow	Smooth	Brown	0.0	0.0	0.0	0.0	0.0	0.0
	BH34	Yellow	Smooth	None	0.0	0.0	0.0	0.0	0.0	0.0
**3**–S3	BH35	White	Rough	Brown	13.6±0.28	15.8±0.41	17.1±0.36	22.3±0.51	20.4±0.40	17.66±0.61
	BH36	Creamy	Rough	None	0.0	0.0	0.0	0.0	0.0	0.0
	BH37	White	Smooth	None	0.0	0.0	0.0	0.0	0.0	0.0
	BH38	White	Rough	Orange	0.0	0.0	15.0±0.20	19.1±0.36	0.0	13.6±0.41
	BH39	White	Rough	None	0.0	0.0	0.0	0.0	0.0	0.0

Table S3. Cultural characteristics of actinomycete isolate BH35 allowed to be grown on ISP and other growth media.

Media	Growth Rate	Colony Color		Soluble Pigments
		Aerial Mycelium	Substrate Mycelium	
Liquid tryptone yeast extract (ISP – 1)	Moderate	Light gray (ISCC-NBS 264)	Brilliant orange yellow (ISCC-NBS 67)	None
Yeast extract-malt extract agar (ISP – 2)	Abundant	White (ISCC-NBS 263)	Light yellow (ISCC-NBS 86)	None
Oatmeal agar (ISP – 3)	Weak	No sporulation	Light yellowish brown (ISCC-NBS 76)	None
Inorganic salts-starch agar (ISP – 4)	Abundant	White (ISCC-NBS 263)	Dark yellowish brown (ISCC-NBS 78)	None
Glycerol asparagine agar (ISP – 5)	Moderate	White (ISCC-NBS 263)	Light yellow (ISCC-NBS 86)	None
Peptone yeast extract iron agar (ISP – 6)	Abundant	White (ISCC-NBS 263)	Medium gray (ISCC-NBS 265)	Strong brown (ISCC-NBS 55)
Tyrosine agar (ISP – 7)	No growth	None	None	None
Starch-nitrate agar	Abundant	Yellowish white (ISCC-NBS 92)	Dark yellowish brown (ISCC-NBS 78)	None
Czapek–Dox agar	Abundant	White (ISCC-NBS 263)	Light yellowish brown (ISCC-NBS 76)	None
Potato dextrose agar	Abundant	Beige to yellowish-white	Dark yellowish brown (ISCC-NBS 78)	None

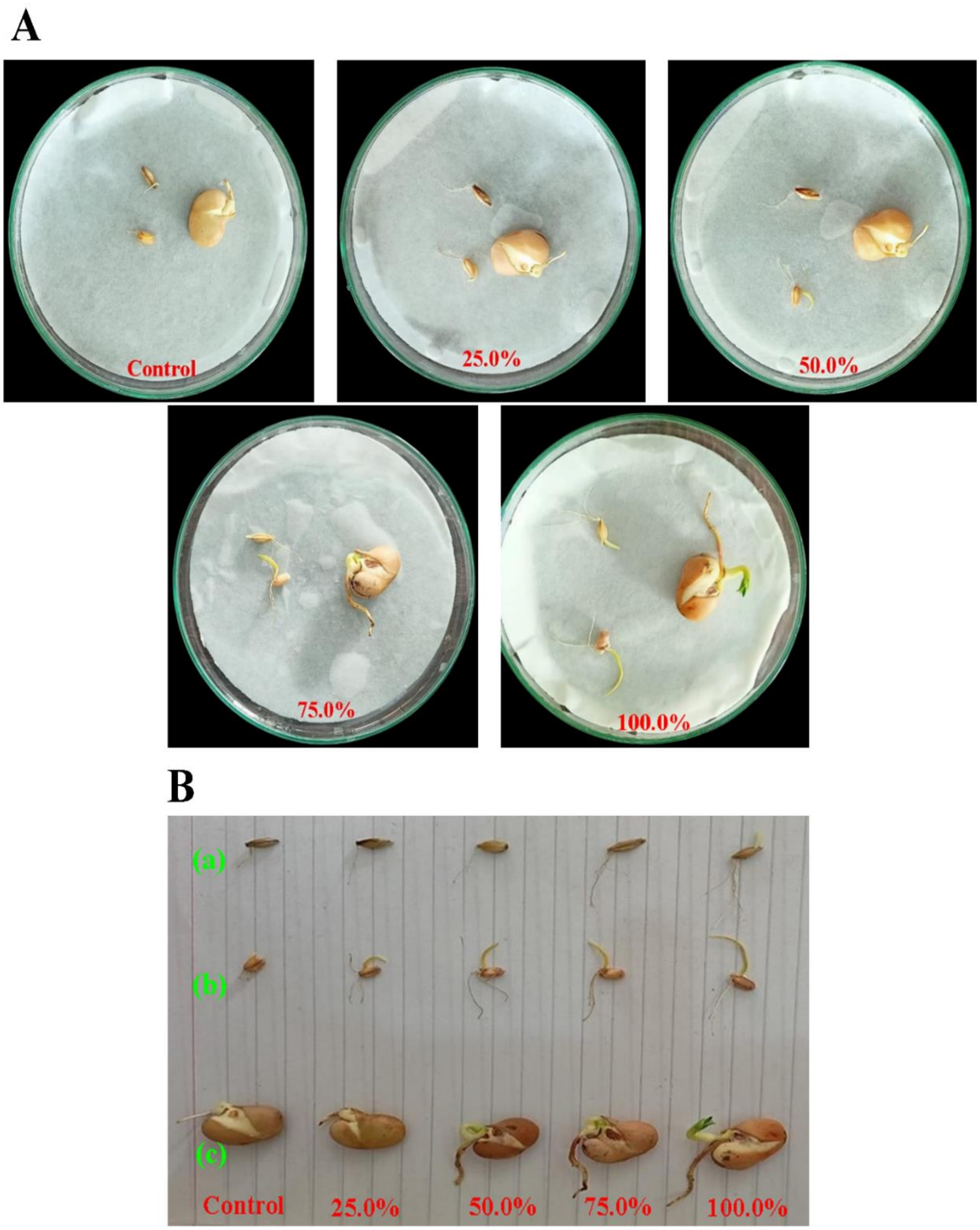

Fig. S1. The promoting effect of different concentrations of CFF of *N. alba* BH35. (**A**) Bioassay of seed germination using filter paper in Petri dishes. (**B**) Stimulating effect on both shoot and root length of the germinated seeds. In this figure (a) Barley (*H. vulgare*), (b) Wheat (*T. sativum*), and (c) Bean (*V. faba*).

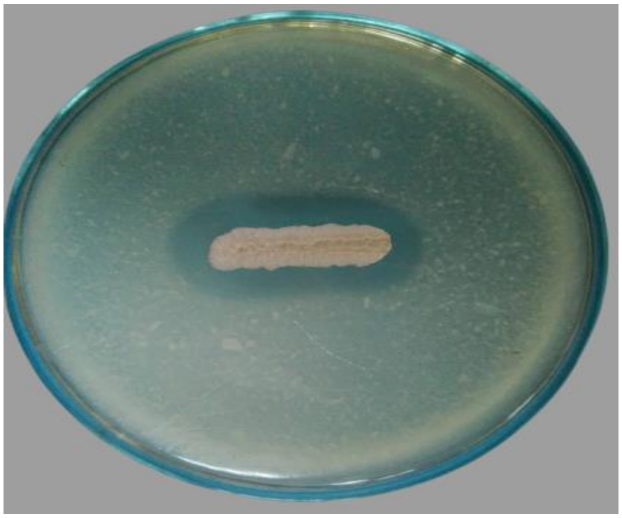

Fig. S2. Phosphate solubilizing by *N. alba* BH35.
